# Refractory and Paradoxical Emesis in a Healthy Psychiatric Patient

**DOI:** 10.7759/cureus.7550

**Published:** 2020-04-05

**Authors:** Iain Curtis-Shanley

**Affiliations:** 1 Emergency Medicine, Montefiore Hospital, New Rochelle, USA

**Keywords:** marijuana, cannabis, cannabinoid hyperemesis syndrome, chs

## Abstract

A woman with a complicated past psychological history presented with a paradox of refractory emesis despite the continual usage of numerous anti-emetic pharmaceuticals. The patient had sought help repeatedly from others in the past. Laboratory results were inconclusive. Eventually, taking a thorough patient history led to the discovery of the obscure reason behind the patient's condition.

## Introduction

Humans have an extensive history of cannabis use. However, the physiological effects of cannabis are still not generally understood by many. The result of this can be catastrophic in individual cases. Marijuana can have anti-anxiety effects, through binding to the cannabinoid receptor type 1 (CB1) receptor in the central nervous system, but can also be a source of mental illness and psychological decompensation, inducing the paradoxic disorders such as anxiety disorder in patients who engage in long-term use of the drug and begin to do so at an early age [1-2]. Cannabis is frequently utilized for its anti-emetic properties, such as in relation to the side effects of chemotherapy, but in a few cases, can cause profound nausea and emesis [3-4].

## Case presentation

Patient is a female in her early thirties with a past medical history of cannabis use disorder, post-traumatic stress disorder (PTSD), depression, pancreatitis, and gastroesophageal reflux disease (GERD), who presented to the ER with the chief complaint of non-bloody emesis for the prior 4 days, at least 5 times a day. The patient also attested to diaphoresis, chills, and nausea. On the day when the patient presented to the ER, the patient described the vomitus as being yellow-green in color.

The patient has a past medical history of PTSD, panic disorder associated with agoraphobia, endometriosis, colitis, gastritis, persistent depressive disorder, syncopal episodes, migraine, GERD, cannabis abuse, vitamin D deficiency, alcohol (ETOH) abuse disorder, gastric outlet obstruction, adjustment disorder with mixed disturbance of emotions and conduct, and borderline personality disorder. She had a prior surgical history of cholecystectomy and tonsillectomy several years prior to the current case.

The patient's medications included Quetiapine (100 mg qd), Pantoprazole (40 mg qd), and Ondansetron (4 mg qd). The patient did not seem to possess any significant allergies, attesting only to fexofenadine (which caused hives) and watermelon (which caused swelling), neither of which were considered to be likely involved in the patient's current presentation.

The patient did possess an extensive history of substance abuse. This was remarkable as her appearance and manner gave no suggestion of this. She attested to using marijuana 4-5 times a week for about 5 years and drinking a pint of vodka 4 times per week for an extensive but unspecified period of time prior. Her family history was not considered significant, consisting only of a paternal history of diabetes and a brother who had a medical history of migraines.

On examination, her vital signs were all within normal limits, and her physical examination (including bowel auscultation) failed to present any significant or unusual findings. The patient's laboratory results were similarly non-significant.

Upon further questioning, the patient stated that the consumption of marijuana improved her nausea. When asked specifically, the patient stated that hot showers did indeed improve her symptoms and that she in fact used these therapeutically to treat her nausea, although she stated that she didn't understand why it was this worked.

## Discussion

Attitudes and beliefs on controversial subjects are rarely uniform in any society, during any period of time. There can be a tendency in periods of social contention to engage in psychological “splitting” and to minimize or ignore the negatives or positives of a thing in contention, depending upon the individual's view of it.

Marijuana's transformation into an illegal substance in the beginning of the twentieth-century led to an emphasis on the negative effects of it. More recent campaigns to legalize the substance have lead to an emphasis on a strictly positive narrative concerning the substance's effect.

However, as Paracelsus remarked, “Sola dosis facit venenum”, or, “the dose makes the poison”. If patients, and practitioners, have a substance represented to them as entirely benign, there is consequently a very low index of suspicion towards the substance as a cause of any complaint. In the case of cannabinoid hyperemesis syndrome, both patient and practitioner will note the existence of marijuana usage, but will likely only interpret it as having anti-emetic properties, and so be puzzled as to why the patient is presenting with nausea. Practitioners and patients will reasonably conclude that marijuana usage is having a positive effect and encourage its continued use, particularly since the patient will likely attest to it providing some relief to their symptoms.

However, what the patient is actually suffering from stems precisely from what they believe is giving them relief. The patient in this case is the victim of a rare, and only recently documented, syndrome: “cannabinoid hyperemesis syndrome”. Contrary to the patient's initial belief, the best treatment is marijuana cessation, which will lead to a swift improvement of a patient's symptoms, often in only a few days [5].

The patient was educated as to the nature of her condition. She consented to continued abstinence from marijuana. After a few days of cessation of the substance, she recovered completely, and required no further anti-emetic medication, or self-medication through any illegal substance.

This case, it may further be noted, was remarkable for an additional reason: the average duration of cannabis abuse before the onset of cannabis hyperemesis disorder is 19 years, with a standard deviation of about 4 years [6]. In this case, the patient had only used cannabis for about five years. This particular case demonstrates that the time requirement of chronic cannabis abuse for this syndrome can actually be significantly shorter than previous estimates have suggested.

## Conclusions

Doctors must strive to provide accurate and thorough education to the general population, especially during periods of social stress and mutability. Patients must be made aware of the sometimes paradoxical effect of marijuana on individual patients, and thus, through a quick and cheap method such as education, side-effects and potential hospital admissions can be prevented.

When an individual has a substance presented as entirely benign, they will have no reason to even begin to suspect it when side effects ensue. A poison that is unsuspected may prove to be the most pernicious.
